# Kawasaki disease in Sicily: clinical description and markers of disease severity

**DOI:** 10.1186/s13052-016-0306-z

**Published:** 2016-11-02

**Authors:** Maria Cristina Maggio, Giovanni Corsello, Eugenia Prinzi, Rolando Cimaz

**Affiliations:** 1 University Department Pro.Sa.M.I. “G. D’Alessandro”, University of Palermo, via dei Benedettini n.1, 90134 Palermo, Italy; 2 NEUROFARBA Department University of Florence, and AOU Meyer, Florence, Italy

**Keywords:** Kawasaki Disease, Vasculitis, Cardiovascular Disease, Small Vessel Vasculitis

## Abstract

**Background:**

Kawasaki disease (KD) is an acute systemic vasculitis of small and middle size arteries; 15-25 % of untreated patients and 5 % of patients treated with intravenous immunoglobulin (IVIG) develop coronary artery lesions (CAL). Many studies tried to find the most effective treatment in the management of resistant KD and to select the risk factors for CAL.

Our data are assessed on children from west Sicily, characterized by a genetic heterogeneity.

**Methods:**

We studied the clinical data of 70 KD Sicilian children (36 males: 51 %; 34 females: 49 %), analysed retrospectively, including: demographic and laboratory parameters; echocardiographic findings at diagnosis, at 2, 6 and 8 weeks, and at 1 year after the onset of the illness.

**Results:**

Forty-seven had Typical KD, three Atypical KD and twenty Incomplete KD. Age at the disease onset ranged from 0.1 to 8.9 years. IVIG were administered 5 ± 2 days after the fever started. Defervescence occurred 39 ± 26 hours after the first IVIG infusion. Fifty-six patients (80 %) received 1 dose of IVIG (responders); 14 patients (20 %) had a resistant KD, with persistent fever after the first IVIG dose (non responders). Ten (14 %) non responders responded to the second dose, 4 (5 %) responded to three doses; one needed treatment with high doses of steroids and Infliximab.

Cardiac involvement was documented in twenty-two cases (eighteen with transient dilatation/ectasia, fifteen with aneurysms). Pericardial effusion, documented in eleven, was associated with coronaritis and aneurysms, and was present earlier than coronary involvement in seven.

Hypoalbuminemia, D-dimer pre-IVIG, gamma-GT pre-IVIG showed a statistically significant direct correlation with IVIG doses, highlighting the role of these parameters as predictor markers of refractory disease. The persistence of elevated CRP, AST, ALT levels, a persistent hyponatremia and hypoalbuminemia after IVIG therapy, also had a statistical significant correlation with IVIG doses.

Non responders showed higher levels of D-dimer and gamma-GT pre-IVIG, persistent high levels of D-dimer, CRP, AST, ALT, hypoalbuminemia and hyponatremia after IVIG.

**Conclusions:**

This is the first study on KD in Sicily. We suggest some laboratory parameters as predictive criteria for resistant KD. Patients who show early pericarditis need careful surveillance for coronary lesions.

## Background

Kawasaki disease (KD) is an acute systemic vasculitis, affecting small and middle size arteries, occurring mainly in infants and children up to 5 years of age [1, 2]. It is the second most common cause of vasculitis after Schönlein-Henoch Purpura and the first cause of acquired heart disease in childhood in western countries [3]. It is self-limited, with fever resolving after an average of 12 days even without treatment [1], of unknown aetiology and is characterized by a marked increase of serum levels of proinflammatory cytokines and chemokines [1] and the activation of the innate and adaptive immune system [4–6].

Timely administration of IVIG is fundamental to reduce inflammation of coronary artery walls and to prevent coronary damage; the long-term treatment in individuals developing coronary aneurysms must prevent myocardial ischemia and infarction [1]. About 15-25 % of untreated KD patients and approximately 5% KD patients who received intravenous immunoglobulin (IVIG) treatment may develop coronary artery lesions (CAL).

IVIG administered 10 days or more after fever onset may contribute to reduce inflammation but are usually thought to be ineffective in preventing CAL [7]. The rate of IVIG resistance is higher in patients treated before the fifth day of fever, though it is unclear whether a poor outcome follows the early treatment or if patients diagnosed with KD before the fifth day have a more severe form of the disease with a higher risk of cardiac lesions [8].

A single high dose of IVIG (2 g/kg), combined with acetylsalicylic acid, is the gold standard therapy in the acute stage of KD. However, approximately 8-38 % of children are unresponsive to initial high dose IVIG treatment and this condition is defined as “refractory KD”. These patients have an increased risk of CAL development.

Many authors tried to define risk factors of IVIG failure. Kuo et al. showed that pre-IVIG hypoalbuminemia (≤2.9 g/dL) was more common in non responders to IVIG than in good responders [9]. Kobayashi et al. defined a score system to calculate the risk of refractory KD. They indentified seven variables to generate a scoring model useful to predict IVIG resistance: days of illness at initial treatment ≤ 4, age ≤ 12 months at the onset, high serum levels of AST ≥100 IU/L, high CRP (≥10 mg/dl), elevated percentage of neutrophils (≥80 %), low serum sodium levels (≤133 mmol/L), low platelet count (≤30.0 × 10^4^/mm^3^) [10]. Other authors included a lower lymphocytes count in the Kobayashi score to increase the predictive value of IVIG-resistant patients during the subacute phase [11]. However the score has not performed well in non-Japanese populations [12].

Failure of the first dose of IVIG remains the most significant risk factor for CAL. However, there are few studies on non-responders to a second dose of IVIG. A recent study reported that patients with CRP level ≥ 8mg/dl after the first dose of IVIG, have a higher risk to fail additional IVIG doses and may require further IVIG plus rescue therapy [13]. Furthermore these patients have a higher incidence of CAL with persistent lesions [13]. Other authors have recently evidenced that elevated D-dimer levels are a risk factor for CAL in patients non responder to IVIG treatment [14]. Recently, fractional change-C-reactive protein was also considered to be useful for predicting initial IVIG resistance in KD [15]. Refractory KD may require additional therapy such as a second dose of IVIG, steroids, infliximab or other biological drugs. As for corticosteroids, Ogata et al. evaluated the efficacy of steroids plus IVIG treatment for KD defined at high risk. Intravenous methylprednisolone at the dose of 30 mg/kg for three days in association with IVIG was effective to guarantee a good response and prevent CAL [16].

We aimed to describe clinical characteristics and severity factors in KD patients in Sicily and to analyze the risk factors of severe and/or refractory form of KD. Sicilian Caucasian population had origin from a miscellaneous of many populations coming from Greece, Northen Europe, Arabic countries, Spain, France, Northen Italy… They conquered this country in the course of the centuries, with complex cultural, familial and genetic interactions. These people married with local people, acquired local customs and traditions and several times maintained their residence in Sicily.

To our knowledge this is the first KD study in the island.

## Methods

### Patients

We analyzed the clinical data of 70 KD Sicilian children, admitted to the Paediatric Clinic of Palermo from 2008 to 2014.

Charts were analyzed retrospectively and data were collected in a customized database. Data included demographic and laboratory parameters (age, sex, days of fever at the admittance, days of fever at the start of IVIG, neutrophils, lymphocytes and platelet count, haemoglobin, albumin, ALT, AST, gamma-GT, C-reactive protein (CRP), erythrocyte sedimentation rate (ESR), Na, D-dimer), as well as echocardiographic findings at diagnosis, at 2 weeks, at 6 to 8 weeks, and at 1 year after the onset of the illness.

More frequent echocardiographic evaluation was performed only in patients at high risk (children persistently febrile or with CAL, ventricular dysfunction, pericardial effusion, or valvular regurgitation).

The study has been granted from requiring ethics approval by the ethics committee. “Comitato Etico Palermo1”.

Ethics committee approval was not necessary since for anonymized data this is not required by Italian regulations.

In order to early identify non responders, after considering the Kobayashi score system and other side factors considered in the international literature, we analyzed the following parameters: age, gender, days of the disease at IVIG start, neutrophils, lymphocytes and platelet count, haemoglobin, albumin, ALT, AST, gamma-GT, CRP, ESR, Na, D-Dimer. We correlated these parameters to each other and with the days of fever at the start of the IVIG treatment, the response to IVIG, the clinical and laboratory outcome, cardiologic involvement and CAL.

### Statistics

All variables were tested for normality with the Anderson-Darling normality test. All variables were expressed as mean ± standard deviation. The degree of linear relationship between clinical, microbiological and biochemical parameters was calculated using Pearson product moment correlation coefficient; statistical significance was accepted at *p* less than 0.05. Calculations were performed using MiniTAB release 13.1 Statistical Software.

## Results

Among the 70 patients [Males: 36 (51%); Females: 34 (49%); age (mean ± SD): 2.17 ± 1.80 years] the male to female ratio of KD patients was 1:1. Forty-seven (68%) were diagnosed as typical KD, 3 (4%) as atypical KD, 20 (28%) as incomplete KD, following the definition of Committee on Rheumatic Fever, Endocarditis, and Kawasaki Disease, Council on Cardiovascular Disease in the Young, American Heart Association [1, 17].

At the hospitalization fever had been present for a mean of 5 ± 2 days; the most frequent signs/symptoms at diagnosis are described in Table 1. Associated findings included radiographically confirmed pneumonia in 6 patients; hepatomegaly documented by ultrasound in 44 patients, and gallbladder enlargement in 35.

**Table 1:** Signs/Symptoms at diagnosis

Symptoms	Number of patients	Percentage (%)
Fever	70	100
Conjunctivitis	62	86
Stomatitis	59	84
Rash	50	71
Lymphadenopathy	43	61
Extremity changes	7	10

Echocardiographic follow up showed the persistence of coronary lesions (varying from coronary brightness to aneurysms), at 6 to 8 weeks, and at 1 year after the onset of the illness in thirty-three patients (47 %): the z-score was > 4 in 15/33 patients (45 %); > 2.5 and < 4 in 18/33 children (55 %). CAL were reported in 22/47 patients (47 %) with typical KD, 2/3 patients (67 %) with atypical KD, 9/20 patients (45 %) with incomplete KD. Among the patients who developed coronary involvement, 25 (76 %) were responders, 8 (24 %) were non responders. Among the 15 patients (21 %) who developed coronary aneurysms, 12 (80 %) had typical KD; one had atypical KD; two had incomplete KD.

IVIG were administered 5.3 ± 2.4 days after the fever started. Defervescence occurred 39 ± 26 hours after the first IVIG infusion. Among the 70 patients, 56 patients (80 %) received 1 dose of IVIG (responders); 14 patients (20 %) had a resistant KD, with persistent fever after the first IVIG dose (non responders). In the group of non responders KD, 10 (14 %) responded to the second dose, 4 (5 %) responded to three doses while only one patient needed treatment with high doses of steroids and Infliximab.

Seventy-one percent (10/14) of non-responders vs 50 % (28/56) of responders showed cardiac involvement (*p* < 0.05). Pericarditis was present in eleven and associated with coronaritis in 7/25 (28%), with aneurysms in 5/15 (33 %), and in seven it was documented earlier than coronary involvement. The evidence of early pericarditis had a significant positive correlation with the development of aneurisms (*p* = 0.035; r = 0.253).

Acquired valvular insufficiency (of tricuspid and/or mitral valves) was documented in 4 patients, all in the non responder group. Valvular insufficiency had a statistically significant positive correlation with the doses of IVIG (*p* = 0.002; r = 0.358) and fibrinogen pre-IVIG (*p* = 0.048; r = 0.273).

Among the 15 patients who developed coronary aneurysms, 11 (73 %) were responders, 4 (27 %) were non responders: these received three IVIG doses.

The presence of coronary aneurysms showed a statistically significant positive correlation with IVIG doses (*p* = 0.026; r = 0.265), with platelet count pre-IVIG (*p* = 0.045; r = -0.240), AST and ALT post-IVIG (*p* = 0.002; r = 0.364 and *p* = 0.001; r = 0.390), gamma-GT post-IVIG (*p* = 0.030; r = 0.290), D-dimer post-IVIG (*p* = 0.023; r = 0.379).

The significant differences between responders and non responders are shown in Table 2.

**Table 2:** Clinical features and laboratory findings in responders and non responders patients

	Responders	Non responders	P
Cardiac involvement	50 % (28/56)	71 % (10/14)	p < 0.05
Age	2.34 ± 1.92 years	2.17 ± 1.80 years	p = NS
First dose of IVIG < 5 days	5/56 (9 %)	4/14 (29 %)	p < 0.05
Hyponatraemia (< 133 mEq/L)	23/56 (41 %)	11/14 ( 79 %)	p < 0.05
D-dimer (ng/ml) pre-IVIG	818 ± 666	1342 ± 898	p < 0.05
D-dimer (ng/ml) post-IVIG	635 ± 545	1073 ± 1011	p < 0.05

Furthermore in the non responder group, 7 (50 %) were males, and 3/7 (43%) did not respond to the second dose of IVIG and received 3 doses of IVIG; 7 non responders (50 %) were females, and only one of them (14 %) did not respond to the second IVIG dose.

We applied the Kobayashi score to our patients and verified that in IVIG non responder group the score was ≥ 4 only in 8/14 patients (57 %). The Kobayashi score was ≥ 4 in 8/15 (53 %) patients who developed coronary artery aneurysms. In IVIG responders, the Kobayashi score was ≥ 4 in 8/54 (15 %).

Natraemia and albuminemia pre-IVIG were lower in non responders, however the correlation did not reach the statistical significance. On the contrary, persistent hyponatraemia and hypoalbuminemia after the first IVIG dose had a significant correlation with the number of IVIG doses, with a negative correlation of IVIG doses with natraemia and albuminemia post-IVIG (*p* = 0.039; r = -0.258 and *p* = 0.05; r = -0.243 respectively), gamma-GT pre-IVIG (*p* = 0.014; r = -0.314), AST post-IVIG (*p* = 0.016; r = -0.290), ALT post-IVIG (*p* = 0.000; r = -0.468), low haemoglobin levels and platelet count after the first dose of IVIG (*p* = 0.05; r = -0.236); it showed a positive correlation with CRP levels post-IVIG (*p* = 0.030; r = 0.263) and D-dimer pre-IVIG (*p* = 0.005; r = 0.389),. However only 4/14 (29 %) in the non responder group had low platelet count in acute phase (≤30.0 × 10^4^/mm^3^).

Coronary aneurysms showed a statistically significant positive correlation with IVIG doses (*p* = 0.026; r = 0.265), with platelet count pre-IVIG (*p* = 0.045; r = -0.240), AST and ALT post-IVIG (*p* = 0.002; r = 0.364 and *p* = 0.001; r = 0.390 respectively), gamma-GT post-IVIG (*p* = 0.030; r = 0.290), D-dimer post-IVIG (*p* = 0.023; r = 0.379).

Furthermore valvular insufficiency had a significant positive correlation with fibrinogen pre-IVIG (*p* = 0.048; r = 0.273).

D-dimer had a statistically significant correlation with several laboratory parameters. D-dimer post-IVIG had a statistically significant negative correlation with CRP post-IVIG (*p* = 0.003; r = -0.475), and a positive correlation with leukocyte count post-IVIG (*p* = 0.004; r = 0.469) and with the percentage of neutrophils post-IVIG (*p* = 0.002; r = 0.493). Fibrinogen pre-IVIG had a statistically significant positive correlation with leukocytes pre-IVIG (*p* = 0.002; r = 0.453) and with the percentage of neutrophils pre-IVIG (*p* = 0.000; r = 0.496).

## Discussion

The aetiology of KD remains unknown; however, epidemiologic evidence suggests the possible role of infectious agents in patients with a genetic background [18]. The incidence of cardiac involvement varies between different studies; however, it is higher in patients with refractory KD and in untreated patients (25%) [19]. In our population coronary artery aneurysms were present in 21% of patients: the majority of them were patients with typical KD. Cardiac involvement was present in 71% of non responders vs. 50% of responders, in accordance with published data [1, 3, 14].

A recent report described patients with precocious pericardial effusion and later development of CAL [20]. In our patients pericardial effusion was associated with coronaritis in 28 %, associated with aneurysms in 33%, and was documented earlier than coronary involvement, confirming previous findings.

Hepatomegaly and gallbladder enlargement were signs documented by ultrasound in 63 % and 50 % of patients, respectively. The high incidence of these complications may be explained by the routine abdominal ultrasound performed in these patients, at diagnosis and during persistence of fever and/or of increased biochemical signs of inflammation.

We tested the ability of the Kobayashi Score [10] to predict IVIG resistance and coronary artery aneurysms in our patients; however these conditions were not predicted by a high Kobayashi Score, because about 50 % of the patients were missed by the score. Other authors tested this and other scores in their populations, but did not demonstrate a diagnostic utility [21–24].

In our patients hypoalbuminemia, D-dimer levels pre-IVIG and gamma-GT pre-IVIG showed a statistically significant direct correlation with IVIG doses, highlighting the role of these parameters as possible predictors of refractory KD. Furthermore, patients who show early pericarditis need more careful surveillance for CAL [20] with regard to treatment [25].

In a recent wide-scale study performed in Japan, age, sex, CRP levels, platelet count, lower albumin levels were confirmed to be linked to a higher risk of CAL [26]. In our population coronary aneurisms showed a statistically significant correlation with IVIG doses, platelet count pre-IVIG; AST, ALT, gamma-GT post-IVIG; D-dimer post-IVIG. These data confirm a link between coronary vasculitis and the severity of systemic vascular inflammation and/or extra-cardiac involvement.

Our center is one of the three major pediatric hospitals in Sicily were children affected by Kawasaki disease are treated. However we have not yet the epidemiological data about the real incidence of Kawasaki disease in our region.

A recent study evaluated epidemiology of KD in central Italy: the peak of incidence was in the second year of life, with a mild prevalence of males. The icidence was 17.6:100.000 children under five years. 5.2 % had one or more cardiac complications, 2.6 % had CAL [27].

## Conclusion

In conclusion, this is the first study on KD in Sicily: we described the clinical data and evaluated the correlation of clinical and biochemical data with the response to IVIG in a genetically heterogeneous population. History reveals that Sicily was the theatre of different dominations (from Phoenicians to Greeks, Romans, Arabs, Normans, Bourbons): these populations leaved in the island art, culture and a complex genetic imprinting whose influence is still alive.
